# Identification Tools of Microplastics from Surface Water Integrating Digital Image Processing and Statistical Techniques

**DOI:** 10.3390/ma17153701

**Published:** 2024-07-26

**Authors:** Ewa Dacewicz, Ewa Łobos-Moysa, Krzysztof Chmielowski

**Affiliations:** 1 Department of Sanitary Engineering and Water Management, Faculty of Environmental Engineering and Land Surveying, University of Agriculture in Kraków, Adam Mickiewicz Ave. 24/28, 30-059 Kraków, Poland; 2 Department of Water and Wastewater Engineering, Faculty of Power and Environmental Engineering, Silesian University of Technology, Akademicka 2A Str., 44-100 Gliwice, Poland; ewa.lobos-moysa@polsl.pl; 3 Department of Natural Gas Engineering, Faculty of Drilling, AGH University of Science and Technology, Oil and Gas, Adam Mickiewicz Ave. 30, 30-059 Kraków, Poland; krzysztof.chmielowski@agh.edu.pl

**Keywords:** characteristics of microplastics, digital image analysis, shape descriptors, principal component analysis, water environment

## Abstract

The primary objective of this study was to demonstrate the potential of digital image analysis as a tool to identify microplastic (MP) particles in surface waters and to facilitate their characterisation in terms of 2D and 3D morphology. Digital image analysis preceded by microscopic analysis was used for an exhaustive quantitative and qualitative evaluation of MPs isolated from the Vistula River. Using image processing procedures, 2D and 3D shape descriptors were determined. Principal Component Analysis was used to interpret the relationships between the parameters studied, characterising MP particle geometry, type and colour. This multivariate analysis of the data allowed three or four main factors to be extracted, explaining approximately 90% of the variation in the data characterising MP morphology. It was found that the first principal component for granules, flakes and films was largely represented by strongly correlated with 2D shape descriptors (area, perimeter, equivalent area diameter) and 3D shape descriptors (Corey Shape Factor, Compactness, Dimensionality). Considering the scraps, principal component PC1 was represented by only five of the above descriptors, and the Compactness variable had the largest contribution to principal component PC2. In addition, for granules, flakes and films, a relationship between 2D shape and the colour of their particles could be observed. For the most numerous MP group identified of multicoloured scraps, no such association was found. The results of our study can be used for further multivariate analysis regarding the presence of microplastic floating on the river surface, with a particular focus on particles of secondary origin. This is of key importance for optimising future efforts in conducting small-scale and multidimensional monitoring of and reducing plastics in the aquatic environment.

## 1. Introduction

Microplastic (MP) is a pollutant that, due to its properties, can easily spread in surface waters such as rivers, lakes, seas and oceans, and then accumulate in bottom sediments. It is estimated that the percentage of MP in aquatic environments, such as seas and oceans, is 15% in water and as much as 70% in marine sediments [1]. In studies on the MP content in the Baltic Sea sediments, the occurrence of this pollutant was found in the amount of 76–295 its/kg_dw_, and in terms of the type of MP, fibre and plastic fragments predominated [2]. During other studies in a larger area of the Baltic Sea, it was found that only fibres occur in amounts as high as 55–9226 its/kg_dw_ [3]. As regards polymer types, polypropylene (PP), polyethylene (PE) and polystyrene (PS) were the most common polymers identified in coastal waters or on Baltic Sea beaches [2,3]. PE, PP, PS and expanded polystyrene (EPS), unlike other plastics (PET, PVC), are lighter than water and float on or near the surface. Their positive buoyancy makes them easily transported in water, collected on shorelines and, consequently, washed ashore even on remote beaches [4]. According to Chubarenko et al. [5], under idealised external conditions, an EPS particle needs only one day to traverse the Baltic Sea, while spherical particles can persist on its surface for 10–15 years.

Procedures (still not standardised) for the determination of MPs in surface waters play an important role in their identification. The GESAMP recommendations [6], which relate to the marine environment, are commonly used and refer to counting plastic particles by origin, shape and colour. Primary microplastics are most commonly described as beads and pellets manufactured in sizes smaller than 5 mm. Secondary MPs are formed by fragmentation and degradation of larger plastic components. Most inaccuracies are caused by the determination of the shape of the MP. Shape factors that characterise natural sediments can be used to describe their particles or methods can be used to determine the main dimensions of non-spherical particles [7]. In the case of natural sediments, shape descriptions are mainly based on geometrical figures, i.e., ellipsoid, flattened spheroid, cylinder, square or disk-shaped plates [8,9]. The Corey Shape Factor (CSF) used for natural particles can be used as a representative parameter to describe the dimensionality of MP. The value of the CSF varies from close to 0 (2D plate/disc) to 1 (perfectly rounded 3D sphere), while a CSF of 0.7 is characteristic of naturally worn sediment [10].

The shape categories of microplastics are commonly used in their analysis, but the actual shape of the particles can vary considerably. After all, plastics are susceptible to the processes of wave-induced fragmentation and UV-induced photodegradation [11,12]. MP transformations, which include changes in density and crystallinity due to photodegradation and weathering (known as plastic ageing), can alter the transport and increase the settling rate of the particles [13,14]. Alimi et al. [15] showed that UV-induced degradation led to increased vertical transport of MP in a lake in Quebec. The weathering of 4.5 mm and 0.22 mm disc-sized MP reduced the settling time of the particles from 3 to 2 h and 18 to 8 days, respectively. Waldschläger and Schüttrumpf [7], on the other hand, while evaluating the effect of shape, size and density on sinking characteristics of MPs, determined an 80× higher settling velocity for EPS pellets compared to polyamide fibres.

Biofouling strongly affects the behaviour of MP in the aquatic environment. The plastic particles can aggregate with particles of biological origin, i.e., phytoplankton or live or dead microorganisms which form zooplankton. The phenomenon of biofilm formation on the MP surface results in an increase in specific gravity and, consequently, faster settling in the water column and accumulation in the bottom sediments of lakes and rivers. Hidalgo-Ruz et al. [16] noted that the settling velocity of PS fragments was higher compared to acrylic fibres and polypropylene pellets with increasing biofilm colonisation. Karkanorachaki et al. [17] showed that the phenomenon of microparticle biofouling in the form of films or pellets also leads to an increase in their settling velocity.

Many authors have conducted studies on the effect of the shape, size and density of plastic microparticles on their settling velocity [7,18,19]. Nguyen [19] analysed the free-fall velocity in the standing water column of polystyrene particles with irregular shapes. He found that the irregularity of EPS could slow down the sinking of large particles, but for too high a level of irregularity, the positive correlation between settling velocity and EPS size became insignificant. The fragmentation and photodegradation processes of expanded polystyrene is an important issue addressed by the researchers. This is because it is one of the most widely used plastics and one of the predominant waste polymers found in the natural environment. Sarkar et al. [12] found that PS particles with an average size of approx. 90–100 µm under optimised atmospheric conditions were broken down into millions of 1–3 µm particles in less than 16 h. According to these authors, the optimised laboratory conditions corresponded to approximately 10 months of MP weathering in the natural environment. Song et al. [11] showed that, as a result of UV exposure and mechanical fragmentation, the number of fragmented EPS, PE and PP particles increased with decreasing particle size. These authors noted that approximately 80% of the EPS granules fragmented into nanoparticles as a result of abrasion alone. Yao et al. [20] report that the colour of EPS particles changes during the accelerated UV-induced ageing process. This is because colour groups are formed on the EPS molecular chain, which causes yellowing of the plastic surface. Following photo-oxidative degradation, the stability of the molecular structure is destroyed, resulting in a change in the crystallinity and specific surface area of the polystyrene particles. Therefore, EPS particles become potential carriers of toxic chemicals, e.g., heavy metals [21,22], or PAHs [23]. Ho et al. [24] explained the effect of weathering on the sorption behaviour of PS in a system composed of multiple organic solutes. In order to understand their sorption mechanisms and the formation of toxic chemicals, EPS particles were modified by abiotic weathering (photodegradation) and/or biotic weathering (microbial degradation). These authors observed inhibition of microalgae growth in the presence of chemically contaminated polystyrene particles. There are many reports that PS deposition on microalgae results in a decrease in chlorophyll concentration and, consequently, in their photosynthetic activity [25,26,27]. Contaminated with toxic substances, MPs also have negative effects on animals. Plastic particles similar in shape and size to natural particles can indeed be mistakenly ingested by animals, e.g., by water birds [28]. Numerous studies have shown that PS micro- and nanoparticles are ingested by marine organisms, causing adverse effects, i.e., reduced foraging activity [29], inhibition of growth and development [30] or oxidative stress [31].

In order to increase the comparability of studies dealing with the identification of MPs in the aquatic environment, appropriate procedures should be developed with a clear distinction of particle shapes. Current forms of MP determination in fresh surface waters are based on the GESAMP recommendations [6]. Water samples are usually concentrated by filtering large volumes of water through plankton nets. This method, called the reduced-volume method, is most suitable for sampling MPs (PP, PE, PS, EPS) floating on the water surface. These plastics are usually separated from organic contaminants by oxidation with hydrogen peroxide. After filtering the sample, flotation is carried out by adding a saturated high-density solution (NaCl, CaCl_2_, ZnCl_2_) [32]. The method of microplastic extraction as a result of sorting and flotation allows the determination of MPs from 5 to 0.3 mm in size by direct observation with a stereoscopic or digital microscope. Manual classification plays a very important role in this stage of MP determination. This is the phase of visual identification of plastic particles, which are sorted according to their size, shape and colour. However, the manual sorting stage is subject to the so-called own error of the marking person. To prevent the loss of information, many scientists use software to process images captured by a stereo microscope equipped with a camera. Image processing using ImageJ 1.52v or ShapeR (R package version 4.0.3) software is becoming an increasingly common tool for morphological assessment (size, colour, shape) and quantitative evaluation of MP particles [33,34].

In this article, an analysis of images obtained with a digital microscope is proposed for the identification of MP particles. Digital image processing using ImageJ software was used to determine the number of microparticles and their basic morphological parameters, describing size, shape and colour. Implemented image processing procedures allowed the extraction and calculation of 2D and 3D shape descriptors and the Principal Component Analysis (PCA) method helped find relationships between shape and colour MPs.

## 2. Materials and Methods

### 2.1. Analysed Samples

A stagnant area of the Vistula River in the north-eastern district of Kraków, below the area exposed to anthropogenic pollution, i.e., urban run-off, was selected for the study. The stagnant area is located on the outskirts of the city, behind the industrial zone, at the marina of Yacht Klub Polski Kraków in the Nowa Huta district (50°03′04″ N 20°03′29″ E). The selection of the sampling site was guided by the location upstream of the treated sewage discharge point and the accessibility of the coastal zone. Additionally, the presence of protected waterbird species was taken into account. Many years of observations by the authors [35] confirm that a marina with peers is a place where mute swans and mallard ducks frequent.

### 2.2. Sampling and Preliminary Sample Preparation

Due to the absence of standardised methods for the collection and separation of microplastics from riverbank sites, the procedures were based on literature reports [36,37,38]. In order to obtain a more representative view of microplastic contamination of the Vistula river, samples were collected for 6 months in the period from March to August 2022.

Water was collected using a reduced-volume method, with a 250 µm mesh size plankton net. On site, 15 dm^3^ of water was passed through the mesh. Samples with microplastics were transported to the laboratory in sealed glass containers.

The analysis of the samples was each time preceded by a pre-treatment to remove mineral and organic contaminants. The material retained on the filter was quantitatively transferred to a laboratory beaker, where natural organic matter was removed. The separated material was rinsed with distilled water and subjected to chemical oxidation using 30% H_2_O_2_, which was repeated if necessary. A density difference method with a 10% NaCl solution was used to separate MP particles [37]. The mixture was allowed to stand for 24 h to allow separation of the plastic particles, and the supernatant liquid was filtered through a GF/A filter (diameter 47 mm, pore size 1.6 μm, Whatman).

### 2.3. Digital Microscopy

Despite the chemical oxidation treatment, organic contaminants, i.e., undegraded plant parts or zooplankton, remained in the samples along with MP. This type of material, which was clearly distinguishable from microplastic particles, was removed during initial sorting under a G1200 digital microscope (Shenzhen Ltd., Shenzhen, China) at 10× magnification.

For the actual image microscopy, a digital microscope was used at 10× to 100× magnification. When visual observations were made, particles with a specific shape (ideally spherical) or characterised by irregular sharp edges were considered plastic particles [16]. Unnatural colour or multicolour also indicated MP particles. A total of 1603 plastic particles were photographed and subjected to further image processing.

### 2.4. Image Processing and Analysis

**Figure 1:**
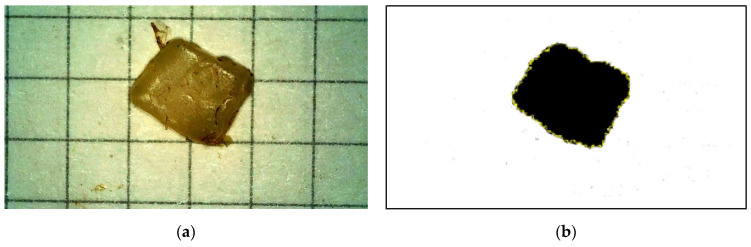
Original photograph of the MP particle (**a**) and its binary image in ImageJ (**b**); grid 2.5 × 2.5 mm.

For each MP particle, images were taken showing their size, shape and colour. The images were then appropriately transformed using open-source ImageJ software (type 1.53a). An example of a binary MP image is presented in Figure 1.

The binary images of MP particles obtained in this way first allowed the determination of two shape descriptors in the 2D plane: surface area (A) and perimeter (P). These parameters were calculated in pixels, which were then converted into units, mm^2^ and mm, respectively.

Based on A and P, a third 2D shape descriptor was determined for each particle image. For the evaluation of pellets, granules, fragments and foils, the area-equivalent diameter (DA) was used. This is calculated by converting the captured image into a circle of equivalent area [39]. For fibres, the ratio of major axis length (L length) to minor axis length (W width) was determined.

### 2.5. Microplastic Characteristics/Morphology

The isolated microplastic samples were analysed to determine the type of particles by origin, shape and colour. Considering the origin, the plastic particles were divided into primary or secondary. Primary MP comes mostly from domestic and agricultural products (textile fibres, cosmetic additives or fertilisers in the form of pellets). Secondary MP results from the fragmentation of larger pieces of plastic.

Based on the GESAMP recommendations [6], all plastic particles were counted according to the shape category. A distinction was also made between regular (pellets) and irregular particles (granules, fragments, films, fibres). Spherical or almost perfectly spherical particles, which were deformed using the hot needle method, were considered pellets [6,40]. Considering irregular spherical or near-spherical particles that can be partially elastic, they were classified as granules. Fragments were defined as irregularly shaped polymer particles in the form of scraps or flakes derived from the fragmentation of larger objects. They looked as if broken off from a larger piece of hard, more convex pieces (scraps) or flat particles with smooth or angular edges (flakes). Films were identified as flexible flat particles with smooth or angular edges. Fibres were defined as a non-tapered long material with a length much greater than the width.

To obtain as complete a view as possible of the MP shape, parameters defining the particle dimensions were used. Area-equivalent diameter (DA) was used to assess the size of pellets, granules, fragments, flakes and foils [41]. The area-equivalent diameter of a particle is the diameter of the circle which has the same area as that of the particle and was calculated as follows [42]:

DA = √ 4 × A_p_/π (1)

where

A_p_—the area of a circle with the same perimeter.

The maximum DA at 5 mm and the area size at a maximum of 19.63 mm^2^ with an acceptable error of <0.5% were defined. In the case of fibres, length L was used to assess their size, which was determined to be equal to or less than 15 mm.

The length (L) was estimated from the maximum Feret diameter. This is the largest distance between two parallel lines that do not intersect the image:

L = F_max_ (2)

where F_max_—maximum Feret diameter.

The width (W) was determined from a rectangle of length L, which has the same area A as the image [43]. According to the current procedure for fibre determination, the ratio of L to W should not be greater than 3 [41].

The overall shape of the particles is described by parameters such as convexity, circularity and elongation, regardless of whether the perimeter is smooth or intricate. A visual determination was made as to whether a determined MP has a shape that indicates that it is a spatial (3D) or a planar (2D) particle. Following the guidelines of Rosal [44], pellets, granules and scraps were considered 3D particles, while 2D particles included flakes and films. Fibres were considered as 1D particles.

In addition to using the usual shape categories for all MPs, an additional subdivision was carried out on the basis of their geometric shape in 2D, i.e., the 2D projection. It was determined how many particles of a given MP type were labelled with a 2D shape approximating a circle, ellipse, polygon, square, rectangle or triangle.

A more detailed description of the morphological properties of plastic materials can be obtained by combining their size and different shape descriptors in the 3D plane. Three factors were used to describe MP particles: the Corey Shape Factor (circularity descriptor), compactness (form descriptor) and dimensionality (shape descriptor). The Corey Shape Factor (CSF) was defined as [10]:

CSF = c/(√ab)(3)

where

a—longest axis of the particle;

b—intermediate axis of the particle;

c—shortest axis of the particle.

The CSF takes values below 0.5 for particles shaped like flakes or discs (2D particles) or fragments (1D particles). Values close to 1 characterise pellet-, granule- and sphere-shaped circular particles (3D particles).

Compactness (C) was defined as follows [42,45]:

C = A/A_P_ (4)

A compactness value of 0 and 1 defines the particles as 1D and ideally 3D, respectively.

Dimensionality (D) was defined as follows [46]:

D = ∑ L_i_/max L_i_ (5)

where

L_i_—the orthogonal length of the particle, i.e., its length in the i-th orthogonal axis.

D takes values from 1 to 3. Particles with dimensionality values close to 1, 2 or 3 can be characterised as 1D, 2D or 3D particles, respectively.

The labelled MP particles were also counted by colour divided into white, grey, black, red, blue, green, yellow and other (transparent, orange, pink and purple).

The estimated number of MP particles was expressed in units per m^3^ of water.

### 2.6. Statistical Analysis

For all shape descriptors in the two-dimensional and three-dimensional planes, basic descriptive statistics were calculated, i.e., mean, median, min, max and SE.

Due to the variability of MP particles, Principal Component Analysis was used to determine the relationships between their geometric shape in 2D (Shape 2D variable), the parameters describing their size and shape (six 2D and 3D shape descriptors) and colour. By reducing the number of primary variables and replacing them with components that significantly explain their variation, PCA analysis allows the processes and phenomena occurring to be described with the maximum amount of information [47]. The Keiser criterion was used to determine the number of principal components. The final step was a graphical representation of the dataset, where each variable was represented by a vector, and its position and length determined the extent to which the individual variables influenced the principal components. Strong correlation occurred when variables whose vectors had similar lengths were located close together in the same part of the factor area. The arrangement of variables far apart or in different parts of the factor area indicated a lack of correlation.

Statistical analysis was performed using Statistica 13.0.

## 3. Results and Discussion

### 3.1. Characteristics of Microplastics

**Figure 2:**
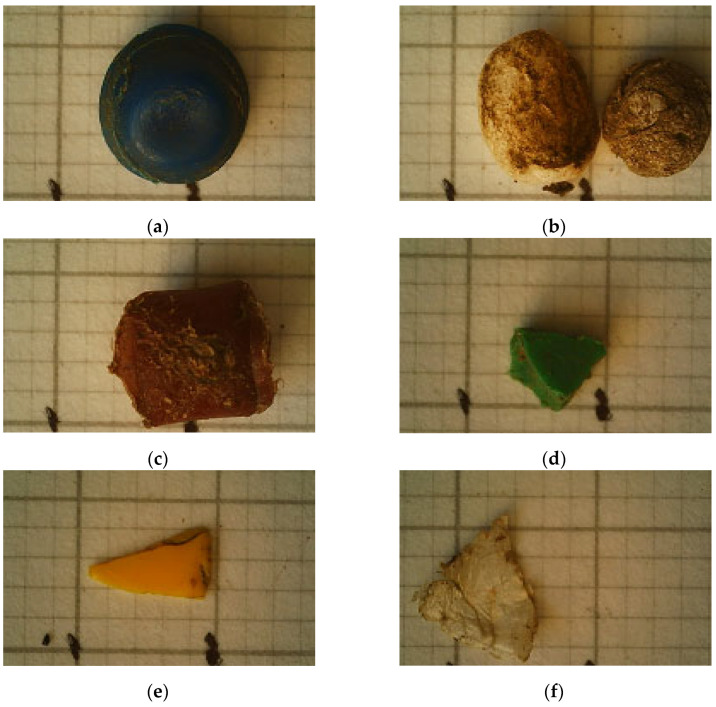
Images of forms of MPs present in the Vistula River: (**a**) disc; (**b**) granules; (**c**,**d**) scraps; (**e**) flake; (**f**) foil by digital microscope; grid 1 × 1 mm.

In terms of the origin of the MP particles, 8933 pcs∙m^−3^ of water were classified as primary. The amount of MPs identified as particles of secondary origin was 97,933 pcs∙m^−3^, representing 91.6% of all plastic particles. This shows that the main source of MPs in the environment is waste from plastic products. The primary sources of MP were mainly household products (microbeads from cosmetics and toy parts in pellet form). The presence of plastic production pellets (so-called nurdles) has also been demonstrated. These are the primary materials for the manufacture of most commercial plastics. Secondary MP resulted from the fragmentation of larger plastic pieces. Figure 2 shows the types of primary and secondary MP retrieved from the Vistula River during the 6-month observation period.

**Figure 3:**
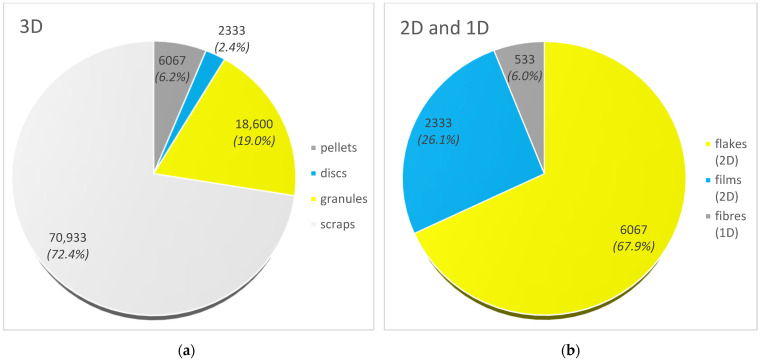
Number of microplastics (items/m^3^) isolated from the Vistula River by shape: (**a**) particles in solid form (3D); (**b**) particles in flat form (2D and 1D).

Based on geometric shape, seven groups of particles were distinguished and divided into 3D particles (regular and irregular) and irregular 2D and 1D particles. Taking 3D particles into account, pellets accounted for 6.2% (6067 items), while granules and trimmings made up 19.0% (18,600 items) and 72.4% (70,933 items) of the designated MP, respectively (Figure 3). Among the regular 3D particles, there was an additional distinction of nurdle, or MP in the form of a flattened ellipsoid (so-called disc), whose content was determined at 2.4% (2333 items). In the case of 2D particles, the content of flakes and films was 67.9% (6067 items) and 26.1% (2333 items), respectively. The content of 1D particles in the form of fibres was the lowest at only 0.5% of all MPs and 6.0% of particles in flat form (533 items).

Table 1 and Table 2 show the morphological characteristics of MP particles, divided into 3D, 2D and 1D particles.

Of the regular 3D particles, pellets had the smallest surface area, circumference and diameter. They were characterised by a diameter of 0.46 ± 0.18 mm. Pellet diameters between 0.2 and 0.3 mm indicated the origin of the particles as a cosmetic additive [48]. Larger diameters indicated the origin of the pellets as, for example, toy parts. The mean surface area and circumference of the pellets were 0.19 mm^2^ and 1.49 mm, respectively. The CSF roundness descriptor of 0.98 ± 0.02 indicated that, among the 3D particles, the form was almost perfectly spherical for all pellets. The compactness and dimensionality values described the form and shape of the pellets as almost perfectly 3D (C~1 and D~3). The low SE values of all shape coefficients indicate the regularity of the pellets. Using the CSF factor, Francalanci et al. [18] and Metz et al. [49] also identified plastic pellets or beads as particles with three dominant dimensions. This confirms that labelled pellets are the primary source of MP in the environment.

The microplastic discs were much larger in size. Compared to pellets, they were characterised by 80× larger surface area, 10× larger circumference and diameter. Their average values were 15.38 mm, 13.93 mm^2^ and 4.43 mm, respectively. The size of the flattened PP pellets studied by Šunta et al. [50] was 4.7 ± 0.1 mm, while Bond et al. [51] report nurdle dimensions ranging from 2.4 mm to 4.6 mm (weathered pellets) and 3.3 mm to 4.0 mm (pristine pellets). The CSF calculated by these authors for the weathered pellets ranged from 0.31 to 0.97 and for all types of pristine pellets from 0.40 to 0.82. These values are similar to the results obtained for the discussed particles. MP in the form of a nurdle was characterised by a circularity descriptor CSF at an average level of 0.84. Based on a CSF close to 1, the discs can be described as 3D particles [10]. However, the values of the other two descriptors C~1 and D~2 indicated that the shape of the nurdles was close to that of 2D particles. Francalanci et al. [18] assigned a 2D shape to the discs. In the present study, the geometric shape of these particles was defined as a flat disk and they were identified as regular 3D particles.

Considering the granules, their diameter was 3.05 mm ± 1.33 mm, while the average area and perimeter were 7.33 mm^2^ and 9.59 mm, respectively (Table 1). Due to the size and elastic nature of the plastic, these particles were probably formed by fragmentation of polystyrene. Kim et al. [52] determined the size of the polystyrene granules to be 3.5 mm ± 0.2 mm, or 15% larger. For the granules in question, the mean CSF value was 0.83. According to Dietrich, a CSF of approximately 0.7 is characteristic of naturally worn sediment [10]. In our study, the CSF values of the granule varied between 0.52 and 0.94, indicating flattened or perfectly spherical particles, respectively. Also, the other two shape form descriptors represented the variable dimensionality of this MP. The values of compactness and dimensionality, ranging from 0.21 to 1.14 and 2.04 to 2.90, respectively, indicated that the granules assumed a 2D or 3D shape. Metz et al. [49] used the term cylindrical granule for particles with 3D dimensions. In the present study, the geometric shape of the granules was defined as irregular 3D particles. This indicates that granules are a secondary source of MP in the environment.

The mean area and perimeter of the scraps were 3.11 mm^2^ and 6.68 mm, respectively. The equivalent surface diameter of these irregular particles was determined to be 2.01 mm ± 1.08 mm. Strady et al. [53] analysing fragments in freshwater in Vietnam estimated the median of their surface area to be 0.13 mm^2^, about 20 times smaller than in our study.

Values of CSF (about 0.2), C (about 0.7) and D (about 2.2) indicated that the geometrical shape of the scraps is close to 2D particles. According to Dietrich, natural particles for which the CSF has a value close to zero are 2D particles that occur in plate form [10]. Francalanci et al. [18] assigned a 1D shape to the fragments, while Metz et al. [49]—a 3D shape. In this study, due to the values of all three coefficients defining roundness, form and shape of the fragments, it was concluded that they should, however, be classified as 2D particles. Their variable geometrical shape confirms the fact that the trimmings are formed by MP fragmentation and are a secondary source in the environment.

Analysing the morphological characteristics of the 2D irregular particles, the mean surface area, perimeter and equivalent surface diameter for the films were found to be larger than for the other irregular particles (Table 2). These were 8.36 mm^2^ ± 4.79 mm^2^, 12.38 mm ± 4.23 mm and 3.21 mm ± 0.93 mm, respectively.

For the flakes, the area and perimeter were determined to be 5.46 mm^2^ ± 3.76 mm^2^ and 8.25 mm ± 4.41 mm. Their equivalent diameter was 2.40 mm ± 1.09 mm. Descriptors defining the geometric shape of the flakes and films (CSF about 0.1, C within 0.6–0.7, D about 2) confirmed their identification as 2D particles. Holjeoć et al. [46] in their study of square plate-shaped MP particles determined D values ranging from 2.17 to 2.33, which confirmed their assumption of classification as 2D particles. According to Metz et al. [49], the term film should refer to particles with two dominant dimensions (2D). A study by Strady et al. [53] showed that the largest fragment area (of the order of 0.3 mm^2^) and its elevated medians were recorded in the waters of Cua Luc Bay.

Of the MPs analysed, fibres had the greatest average length at 6.15 mm ± 4.55 mm. Their surface area and circumference were 2.94 mm^2^ ± 1.38 mm^2^ and 12.30 mm ± 9.09 mm, respectively. As proposed by Francalanci et al. [18] and Metz et al. [49], fibres, i.e., particles with only one dominant dimension were assigned a 1D shape, as indicated by CSF~0 and D~1.

**Figure 4:**
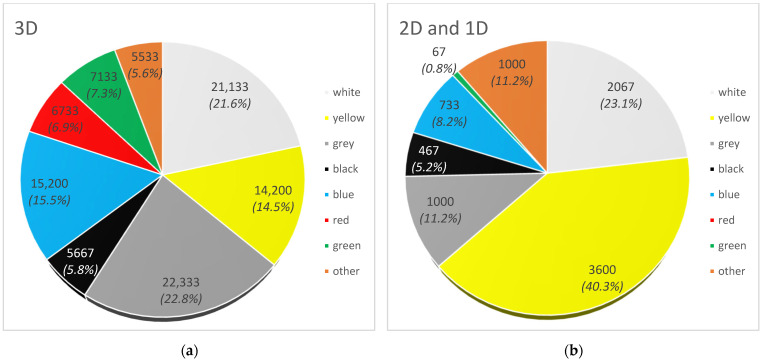
Number of microplastics (items/m^3^) isolated from the Vistula River by colour: (**a**) particles in solid form (3D); (**b**) particles in flat form (2D and 1D).

Eight groups of particles were distinguished by colour (Figure 4). Three-dimensional particles, i.e., in the form of a solid, were mostly characterised by grey (22,333 items—22.8%), white (21,133 items—21.6%), blue (15,200 items—15.5%) and yellow (14,200 items—14.5%) colours. Black, red, green and other colours accounted for 5.8% (5667 items), 6.9% (6733 items), 7.3% (7133 items) and 5.6% (5533 items) of 3D geometric MPs, respectively. Two-dimensional particles were mostly characterised by yellow (3600 items—40.3%) and white (2067 items—23.1%). Grey, black, blue and other colours accounted for 11.2% (1000 items), 5.2% (467 items), 8.2% (733 items) and 11.2% (1000 items) of 2D geometric shape MPs, respectively. Green particles were in negligible quantity (67 items) and red particles were not found. In the case of 1D particles, i.e., fibres, their colour was only grey and black. The fibres determined by Uurasjärvi et al. [54] were characterised by several colours: white, blue, red, brown, black and green. In the case of fibres, it is particularly important to prepare samples for microscopic analysis, because the fibres may be confused with natural plant tissues [55].

The observed different colours of MP particles, among which, grey, white and blue were the most frequently observed, are consistent with reports by other authors [56]. de Calvarho et al. [57] observed similar colours when studying the impact of urbanisation on MP in the Garonne River. White and blue MP represented 32.4% and 14.3% of particles, respectively. In turn, in the lake waters, white MP constituted as much as 80% and blue MP only 8%. The remaining colours red, black/grey, orange and green accounted for less than 4% [58]. Also, in marine sediments from the Black Sea, transparent and white MP was found in the largest amounts [59]. White and blue colours are commonly used in many plastic products, i.e., food packaging, bottle caps and films. The transparent colour is associated with commonly used packaging materials, i.e., bottles and disposable bags. Similar conclusions were reached by Erdoğan [60] when conducting a study on the presence of MP in a pond located in the recreational area of Yozgat Pine Grove National Park in Turkey. Yellow particles were a large group in our study. Their quantity was influenced by the yellowing of EPS granules due to ageing.

### 3.2. Shape 2D and Colour of Microplastics

Given the varying morphological characteristics and colour of the 3D and 2D particles, a particle-by-particle PCA was performed. Due to the negligible content of fibres, they were not included in the analysis.

PCA for granules, flakes and films identified three main factors PC 1, PC 2 and PC 3, which explained about 90% of the variation in the raw data (Table 3). For trimmings, four main factors were determined for which the % cumulative variance was at the highest level of more than 90%. For pellets, the first, second and third principal components explained 48.79%, 76.64% and 90.00% of the total variance of the primary variables, respectively. For the trimmings, PC 1, PC 2, PC 3 and PC 4 explained 51.25%, 68.74%, 81.18% and 93.42% of the total variance. For the flakes, PC 1, PC 2 and PC 3 were 44.67%, 7396% and 87.13% of the total variance of the primary variables, respectively. For films, the first, second and third principal components explained 47.85%, 76.20% and 88.70% of the total variance, respectively.

The PCA performed for the granule showed that the first principal component of PC1 was almost entirely represented by six highly correlated descriptors: a 2D shape (A, P, DA) and a 3D shape (CSF, C, D) (Table 4).

For scraps, five of the above descriptors contributed to PC1, as variable C represented the second principal component. The first component for flakes was shown to be correlated at a high level with six of the shape descriptors, except that variable A made a similarly strong contribution to both PC1 and PC2. Gabrijelčič Tomc and Hladnik [61], in a PCA of the use of 2D shape descriptors in computer simulation of fabrics, found similar relationships. The first principal component was largely represented by the following mutually strongly correlated 2D shape descriptors: area, perimeter and area-equivalent diameter. In the present study, the PCA performed for the films showed that only one 2D shape descriptor (P) and three 3D shape descriptors (CSF, C, D) contributed to the first principal component.

Considering the second principal component of the granule, it was found to be correlated with the Shape 2D and Perimeter variables. For PC2 scraps, it was represented by the form descriptor (C). It was observed that, compared to the other MP particles, this may have been influenced by the more intricate contour of the scraps, affecting the size of their perimeter. As mentioned above for the flakes, PC2 was represented by a variable area. The PC2 of the films was shown to be correlated at a high level with two 2D shape-determining descriptors (A, DA).

It was found that for granules, flakes and films, the variables Colour and Shape 2D were high contributors to the third principal component. These two variables in the case of the scraps decomposed into separate principal components PC4 and PC3, respectively.

**Figure 5:**
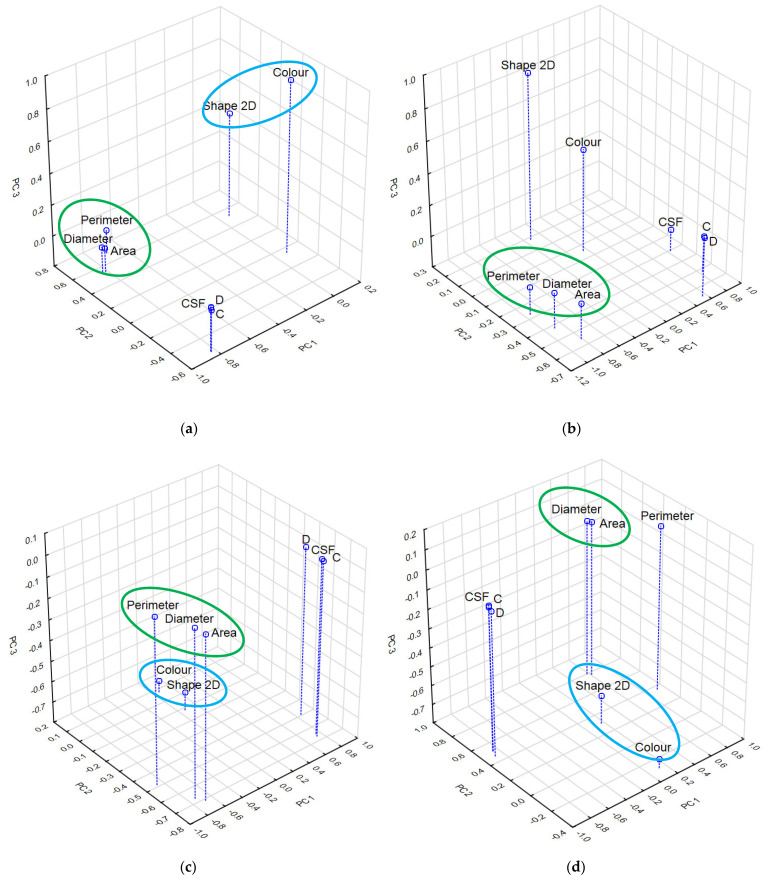
Projection of variables on the principal components area (1 × 2 × 3) for (**a**) granules; (**b**) scraps; (**c**) flakes and (**d**) films.

The correlations between the principal components (PC1, PC2, PC3) and the eight primary variables, i.e., colour, as well as the four 2D shape descriptors (Shape 2D, Area, Perimeter and Area-equivalent diameter) and the three 3D shape descriptors (CSF, Compactness, Dimensionality) are graphically presented in Figure 5 as a projection of variables on the factor area (1 × 2 × 3).

In Figure 5a,c,d, it can be observed that, for granules, flakes and films, the Shape 2D and Colour variables are correlated (marked with a blue ellipse). Such a correlation was not detected for the scraps, as the Shape 2D and Colour variables are located in different parts of the factor area (Figure 5b). The scraps were also found to be distinguished by the different positioning of all the 3D shape descriptors compared to the rest of the MP particles. Two of them, i.e., the circularity descriptor (CSF) and the shape descriptor (D), are located close to each other, while the form descriptor (C) is situated in a different part of the factor area. This may be due to the ambiguous designation of the scraps as 2D or 3D particles due to their variable roundness and form. Li and Iskander [62], when analysing the relationship of nine shape descriptors and six size descriptors for different types of sand, found no correlation between them. Following this, they suggested dividing the descriptors into four independent groups, which included descriptors characterising the overall shape of the particle, perimeter smoothness descriptors, roundness descriptor and convexity descriptor.

Considering the granules, scraps and flakes in question, a correlation can be seen between the three 2D shape descriptors describing their size—the variables Area, Perimeter and Diameter marked with a green ellipse (Figure 5a–c). In the case of the films, a correlation can only be seen between the Area and Diameter variables. This could be due to the intricate contour of these particles, affecting the size of their perimeter. Panunzi et al. [63], in a study on the diversity of MP types consumed by fish, observed differences probably due to different feeding habits. The characterisation of MPs using 2D and 3D shape descriptors allowed a preliminary elucidation of the mechanisms of selection of these particles by marine animals. Using PCA analysis, these authors showed that the ingestion of plastic particles by fish is influenced by their size, circularity and compactness.

**Figure 6:**
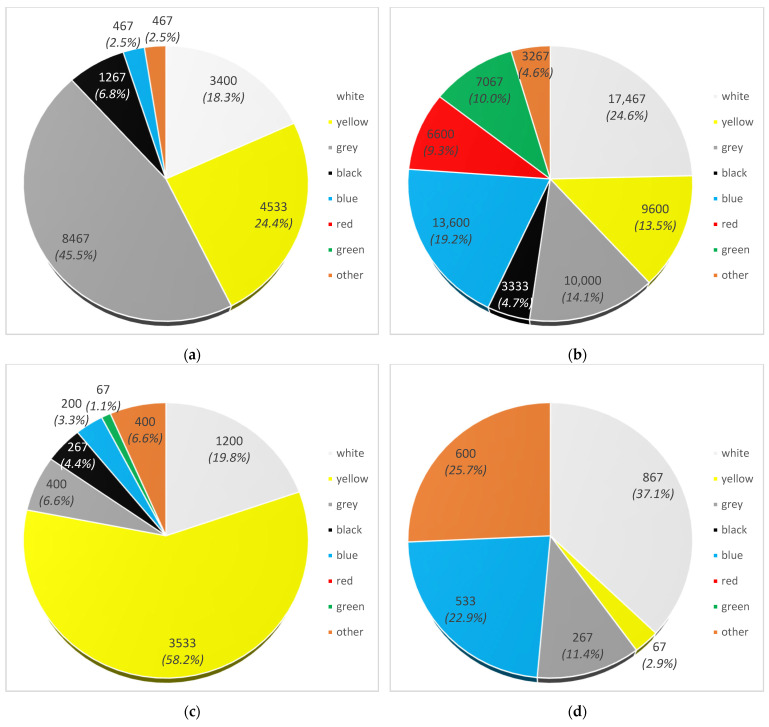
Number of microplastics (items/m^3^) isolated from the Vistula River by colour: (**a**) granules; (**b**) scraps; (**c**) flakes; (**d**) films.

Figure 6 shows the number of individual MPs (granules, scraps, flakes and foils) broken down into the eight differentiated colour groups: white, grey, black, red, blue, green, yellow and other (transparent, orange, pink, purple).

In terms of colour, most granules were identified in grey (8467 items—45.5%), yellow (4,533 items—24.4%) and white (3400 items—18.3%). A total of 6.8% were black granules (1267 items), while the remaining granules were blue (467 items—2.5%) and other, i.e., pink (467 items—2.5%) (Figure 6a). No granules were observed in red and green.

Scraps were most often characterised by white (17,467 items—24.6%), blue (13,600 items—19.2%), grey (10,000 items—14.1%) and yellow (9600 items—13.5%). Green, red, black and other colours accounted for 10.0% (7067 items), 9.3% (6600 items), 4.7% (3333 items) and 4.6% (3267 items) of the total scraps, respectively (Figure 6b). Of the colour differences, orange, pink and purple were identified. Uurasjärvi et al. [54], when determining microplastic in the surface waters of a northern European lake, found that most of the floating fragments found were in white, blue, green and red colours.

For flakes, 58.2% occurred in yellow (3533 items) and 19.8% in white (1200 items). The colours grey, black, blue and green accounted for 6.6% (400 items), 4.4% (267 items), 3.3% (200 items) and 1.1% (67 items) of all flakes, respectively (Figure 6c). The colour other, which was only translucent, highlighted 6.6% of the particles in question (400 items). No red-coloured flakes were identified.

Most foil was identified in white (867 items—37.1%), blue (533 items—22.9%) and other—mostly transparent (600 items—25.7%). A total of 11.4% (267 items) was grey foil and 2.9% (67 items) of the foil was yellow (Figure 6d). No film was observed in black, red or green.

The total amount of blue scraps, flakes and film at 14,800 items (13.8% of all MPs) is a concern, as studies report that fish can selectively digest plastic particles coloured blue [64,65]. Lin et al. in their review showed that fibres and fragments were the most frequently determined forms of MP in fish, at 75% and 18%, respectively. The dominant colours were blue (35%) and black (31%), followed by transparent (19%) and white (13%) [66].

**Figure 7:**
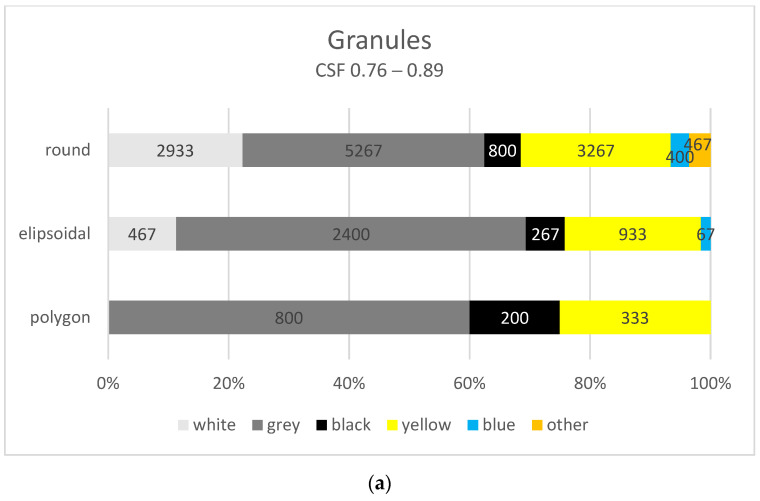

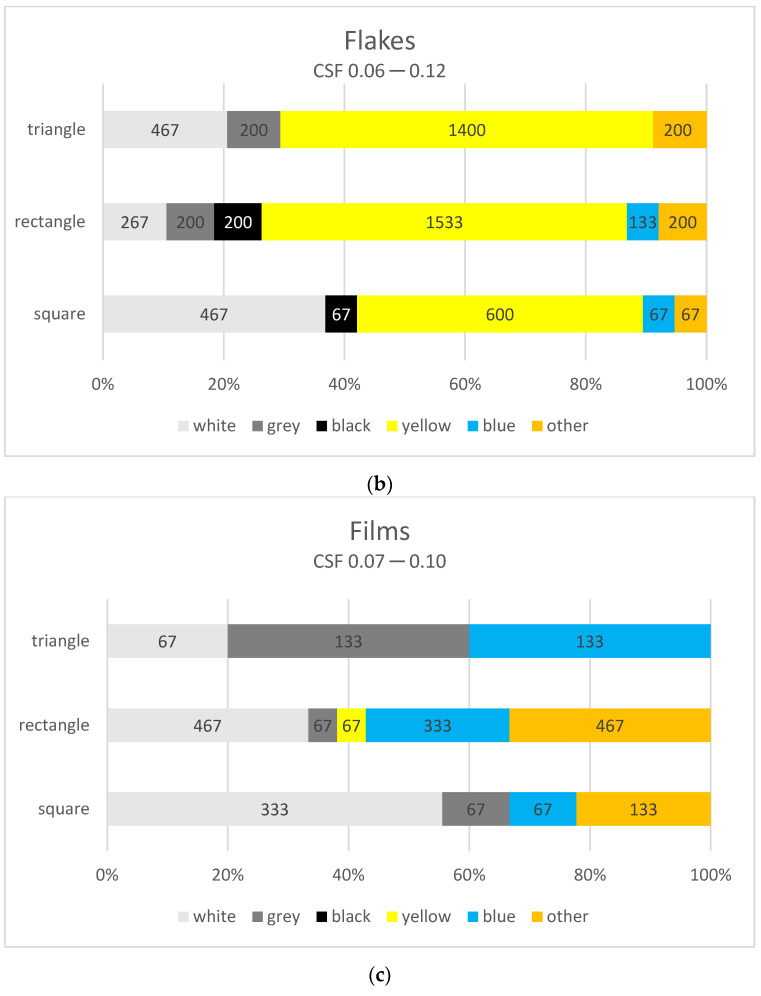
Number of microplastics (items/m^3^) isolated from the Vistula River by Shape 2D: (**a**) granules; (**b**) flakes; (**c**) films.

The PCA showed that, for granules, flakes and films, the Shape 2D and Colour variables are correlated. It is important to remember that plastics change their colour and shape due to, among other things, degradation processes and a reduction in the molecular weight of the polymer. This results in a polymer that is more brittle and thus more susceptible to mechanical and/or biological degradation [67]. Zhu et al. [68] showed that photochemical changes in the properties of plastics occurred as a result of MP irradiation with simulated UV light. These consisted of the white EPS turning yellow and the transparent PP and PE particles turning opaque. After 54 days under simulated sunlight, the EPS, PP and PE particle was reduced in plastic mass. As reported by Chalmers and Meier [69], the white EPS granules under UV light turn yellow after 3 months. Also, their shape changes—initially spherical with a regular surface becoming ellipsoidal at first and then increasingly irregular. However, MP located deep in water or soil is less subject to physicochemical erosion and more to biodegradation processes [70]. Kim et al. [52], who were conducting a study on the shape and morphology of polystyrene MPs, found a lower content of spherical shape particles in the effluent compared to the prolate spheroid (P-spheroid) shape. The aged/weathered EPS particles are characterised by a rough, oxidised surface and heterogeneous morphology, with colour from grey to black. In the study in question, image analysis revealed that 70.6% (13,133 items) of the polystyrene particles were circular in shape with an average CSF of 0.89 (Figure 7a). White, grey and yellow granules accounted for 22.3% (2933 items), 40.1% (5267 items) and 24.9% (3267 items) of the round particles, respectively. The least number of round granules was identified in black (6.1%—800 items), blue (3.0%—400 items) and other, i.e., pink (3.6%—467 items).

The ellipsoid-shaped particles of 4,133 items, which represented 22.6% of the analysed granules, had a CSF at an average level of approximately 0.80. Among the ellipsoidal granules, the grey colour marked 58.1% (2400 items) of the polystyrene particles. Yellow and white colour accounted for 22.6% (933 items) and 11.3% (467 items), respectively. This may indicate the progressive ageing of the polystyrene granules under UV light and their gradual degradation. Black-coloured ellipsoidal particles accounted for 6.5% (267 items) and the fewest particles were identified in blue (1.6%—67 items).

The remaining EPS were considered irregular particles. Their 2D shape was determined to be polygonal, and the average CSF value was found to be the lowest at 0.76. Grey, yellow and black granules accounted for 60.0% (800 items), 25.0% (333 items) and 15.0% (200 items), respectively. No polygonal white granules were observed, indicating that their ageing state was mature due to the degradation processes of the polystyrene particles taking place.

As shown in Figure 7b, the number of rectangular, triangular and square flakes in yellow was highest at 1,533, 1,400 and 600 items, respectively. Grey rectangular and triangular and black rectangular and square flakes were recorded at 200, 200, 200 and 67 items, respectively. This indicates the identification of 69.2% of flakes (4200 items) that may have been fragmented and aged by UV radiation. The white square, triangular and rectangular flakes were 467, 467 and 267 items, respectively. Considering the shape of the flakes, the total content of rectangular, triangular and square particles was 41.8%, 37.4% and 20.9%, respectively. Sorasan et al. [71] highlighted the ageing of PP and PE fragments and EPS granules in the marine environment. Their study showed that the formation of polymeric nanoparticles resulted first from photochemical reactions and then from their mechanical degradation. As was evident from the MP surface texture image, the PP fragments could be split into rectangular, triangular or square particles as a result of UV radiation.

Analysing the different colours of the films, it was found that 60.0% (600 items) of the particles were rectangular in shape, while 25.7% (1,400 items) and 14.3% (333 items) were square and triangular in shape, respectively (Figure 7c). White rectangular, square and triangular foils were identified with 467, 333 and 67 items, respectively. Blue rectangular, triangular and square particles were recorded at 333, 133 and 67 items, respectively. Rectangular and square films in a different colour—transparent—occurred in quantities of 467 and 133 items, respectively. This type of microplastic in grey and yellow was the smallest (267 and 67 items, respectively), and black was not identified. This may be due to other processes occurring during film ageing. Indeed, the morphological changes of flat plastic particles, due to abiotic effects of the environment, are different for flexible particles such as films compared to hard flakes. Julienne et al. [72], analysing the ageing of LDPE films, found that, due to the plasticisation of the polymer, water probably accelerates MP cracking. This occurs in a direction normal to the extrusion direction of the film which causes these flexible plastics to elongate and break with a near rectangular shape.

## 4. Summary and Conclusions

This study confirms the problem of pollution of surface waters by microplastics, in particular polystyrene. In the analysed stagnant Vistula River, located below an area subjected to strong anthropopression, the amount of plastic particles recovered from the river during the 6 months of the study amounted to 106,800 pieces from 1 m^3^ of water. Scraps and flakes accounted for 66.4% and 5.7%, respectively, of the particles extracted from the Vistula River. Their shape and unnatural colour indicated that degraded packaging and bottles were the main sources. Granules, which originated from the fragmentation of polystyrene foam, were also a large group at 17%. It should be remembered that polystyrene, or expanded polystyrene, has one of the highest production rates among plastics. Digital image analysis proved to be an effective tool for the identification of microplastics from the surface water. The publicly available ImageJ programme has enabled accurate morphological characterisation of both spherical and irregularly shaped particles. The 3D properties of MP, which can be described by 2D and 3D shape descriptors, can largely suggest the starting material of the microplastic. Some shapes may be derived from specific products—pellets are likely to be microbeads used in cosmetics, film is mainly derived from plastic bags and packaging materials and granules are derived from polystyrene damage [56].

This article shows that multivariate data analysis using the PCA method provided an opportunity to replace the input set of correlated parameters with three uncorrelated principal factor components, representing linear combinations of variables. It was found that for granules, flakes and films, the third principal component was most related to the variation in the 2D shape and colour of the plastic particles. Such a relationship was not found for the most numerous MP group, i.e., the trimmings, which were described by four principal components. On the one hand, this is related to changes in the colour and shape of the plastic particles due to their degradation processes and progressive fragmentation. On the other hand, the number of colours for granules, flakes and films was not as varied as in the case of trimmings. Indeed, the latter were identified in 10 different colours—white, yellow, grey, black, blue, red, green, orange, pink and violet. Considering the granules, it was concluded that the white particles came from damaged polystyrene food storage containers, beverage cups and insulation materials. The relationships between the shape and colour of the EPS granules indicate their susceptibility to photodegradation processes. The proposed method can be used to track MP transformations in the natural environment and assess the negative impact on aquatic organisms.

In order to reduce the amount and, therefore, the volume of MP in surface waters, there should be awareness-raising campaigns by local authorities about this risk. It is of great importance to publicise this problem, as plastic packaging—especially those used to store food—is very common and its use exposes the environment to years of harmful microplastic particles and is risky to health [73]. It is also important to introduce another stage of municipal sewage treatment, which will limit the release of MP along with the treated sewage.

Multidimensional image analysis using 2D and 3D shape descriptors is a simple and low-cost way to convert categorical data into quantitative variables, avoiding loss of information. A more detailed description of MPs could expand our knowledge of their diversity and distribution in the environment. For this reason, further research will focus on the analysis of black and grey MPs and organic particles. For this purpose, a thermal imaging camera operating in the 400–1000 nm VNIR band will be tested.

The developed method, integrating digital image processing and statistical techniques, can be used in assessing environmental risks after natural or man-made disasters (e.g., as a result of floods). The target group may be state authorities responsible for monitoring the quality of the natural environment.

## Figures and Tables

**Table 1:** Morphological characteristics of 3D particles.

Type of MP	3D Regular Particles						3D Irregular Particles					
	Pellets			Discs			Granules			Scraps		
Parameters describing size/dimension												
Descriptive statistics	A	P	DA	A	P	DA	A	P	DA	A	P	DA
Mean	0.19	1.49	0.46	15.38	13.93	4.43	7.33	9.59	3.05	3.11	6.68	2.01
Median	0.14	1.40	0.43	16.35	14.37	4.57	8.00	10.06	3.20	2.79	6.06	1.88
Min	0.03	0.62	0.20	8.76	10.49	3.34	0.22	1.66	0.53	0.02	0.52	0.16
Max	0.97	3.52	1.11	19.55	15.66	4.99	19.71	15.78	5.01	19.75	16.02	5.02
SE	0.04	0.59	0.18	2.31	1.52	0.47	1.51	5.11	1.33	1.08	2.37	1.08
Parameters describing shape												
Descriptive statistics	CSF	C	D	CSF	C	D	CSF	C	D	CSF	C	D
Mean	0.98	0.94	2.93	0.84	0.99	2.21	0.83	0.83	2.67	0.21	0.71	2.25
Median	0.98	0.96	2.95	0.89	0.99	2.21	0.85	0.87	2.70	0.22	0.73	2.21
Min	0.88	0.68	2.69	0.69	0.92	2.14	0.52	0.21	2.04	0.02	0.19	1.60
Max	1.01	1.01	3.01	0.94	1.04	2.31	0.94	1.14	2.90	0.26	1.04	4.12
SE	0.02	0.08	0.07	0.08	0.03	0.04	0.06	0.16	0.13	0.02	0.15	0.29

A—Area; P—Perimeter; DA—Area-equivalent Diameter; CSF—Corey Shape Factor; C—Compactness; D—Dimensionality.

**Table 2:** Morphological characteristics of 2D and 1D particles.

Type of MP	2D Irregular Particles						1D Particles		
	Flakes			Films			Fibres		
Parameters describing size/dimensions									
Descriptive statistics	A	P	DA	A	P	DA	A	P	L
Mean	5.46	8.25	2.40	8.36	12.38	3.21	2.94	12.30	6.15
Median	3.36	7.38	2.07	8.46	12.93	3.28	3.05	8.99	4.49
Min	0.08	1.05	0.32	1.75	4.87	1.50	0.54	2.71	1.36
Max	19.61	15.76	4.99	19.72	16.01	5.01	4.55	29.01	14.50
SE	3.76	4.41	1.09	4.79	4.23	0.93	1.38	9.09	4.55
Parameters describing the shape									
Descriptive statistics	CSF	C	D	CSF	C	D	CSF	C	D
Mean	0.11	0.69	2.11	0.10	0.59	2.01	0.02	0.48	1.01
Median	0.11	0.70	2.10	0.09	0.63	2.05	0.02	0.55	1.00
Min	0.06	0.19	1.57	0.06	0.25	1.65	0.01	0.07	1.00
Max	0.12	0.95	2.72	0.12	0.87	2.22	0.05	0.93	1.02
SE	0.01	0.14	0.15	0.01	0.14	0.14	0.01	0.34	0.01

A—Area; P—Perimeter; DA—Area-equivalent Diameter; CSF—Corey Shape Factor; C—Compactness; D—Dimensionality.

**Table 3:** Eigenvalues of the correlation matrix (3D and 2D particles by type).

Type of MP	3D Unregular							2D Unregular					
	Granules			Scraps				Flakes			Films		
Principal Component	PC 1	PC 2	PC 3	PC 1	PC 2	PC 3	PC 4	PC 1	PC 2	PC 3	PC 1	PC 2	PC 3
Eigenvalue	3.90	2.23	1.07	4.10	1.40	0.99	0.98	3.57	2.34	1.05	3.83	2.27	1.00
% variance	48.79	27.85	13.36	51.25	17.45	12.44	12.24	44.67	29.29	13.17	47.85	28.35	12.50
% cumulative variance	48.79	76.64	90.00	51.25	68.74	81.18	93.42	44.67	73.96	87.13	47.85	76.20	88.70

**Table 4:** The principal components and factor coordinates of variables (3D and 2D particles by type).

Type of MP	3D Unregular							2D Unregular					
	Granules			Scraps				Flakes			Films		
Variables	PC 1	PC 2	PC 3	PC 1	PC 2	PC 3	PC 4	PC 1	PC 2	PC 3	PC 1	PC 2	PC 3
Colour	0.08	−0.05	**0.90**	0.15	−0.04	0.46	**−0.88**	0.06	0.08	**−0.74**	0.13	−0.29	**−0.75**
2D shape descriptors													
Shape 2D	0.07	**0.50**	**0.50**	−0.14	0.18	**0.87**	0.43	0.06	−0.12	**−0.71**	0.17	0.31	**−0.65**
A	**−0.82**	0.53	−0.04	**−0.81**	−0.55	0.03	−0.02	−0.68	**−0.71**	−0.02	0.58	**0.80**	0.02
P	**−0.70**	**0.70**	−0.04	**−0.94**	−0.27	−0.02	−0.03	**−0.87**	−0.49	−0.01	**0.92**	0.36	0.07
DA	**−0.83**	0.55	−0.04	**−0.90**	−0.42	0.03	0.00	**−0.73**	−0.67	0.00	0.61	**0.77**	0.02
3D shape descriptors													
CSF	**−0.83**	−0.54	0.08	**0.83**	−0.31	−0.06	0.00	**0.73**	−0.67	0.04	**−0.85**	0.51	−0.05
C	**−0.83**	−0.55	0.07	0.55	**−0.66**	0.18	0.15	**0.75**	−0.65	0.04	**−0.85**	0.51	−0.04
D	**−0.83**	−0.54	0.08	**0.84**	−0.52	0.03	0.07	**0.85**	−0.50	0.01	**−0.88**	0.46	−0.05

A—Area; P—Perimeter; DA—Area-equivalent Diameter; CSF—Corey Shape Factor; C—Compactness; D—Dimensionality. The highest correlation is bolded.

## Data Availability

The data presented in this study are available upon request from the corresponding author (due to privacy).
